# Chromosome-Scale Assembly of Novel *Caenorhabditis sp. 61* (strain JU4110)

**DOI:** 10.17912/micropub.biology.002358

**Published:** 2026-09-11

**Authors:** Pooja Lad, Michelle A. McCauley, Victoria K. Eggers, Janna L. Fierst, Karolina Willicott

**Affiliations:** 1 Biological Sciences, Florida International University, Miami, FL, United States

## Abstract

*Caenorhabditis* nematodes are similar morphologically but highly distinct on the genetic level, with recent characterizations being based on DNA sequences. Comparisons between the model *C. elegans* and other *Caenorhabditis* species have revealed conserved chromosome organization and species-species changes that have improved our understanding of genome evolution. Here, we report a chromosome-scale assembly of a novel *Caenorhabditis* *sp.* *61* (strain JU4110). This reference genome expands the genomic resources available for genus *Caenorhabditis* and allows for more detailed comparative analyses and greater phylogenomic coverage.

**Figure 1. PacBio and Hi-C sequencing leads to a chromosome-scale assembly of novel Caenorhabditis sp. 61:** **A.** Snail plot of assembly statistics for JU4110. The outer circumference represents the full length of the genome. The rings, moving from outermost to innermost describe % GC content, N90 length, N50 length, longest scaffold, and scaffold length and number. The top right circle displays BUSCO scores. **B.** Hi-C contact map of JU4110 assembly reveals the six chromosome-scale scaffolds corresponding to the X sex chromosome and autosomes I-V, which are ordered from largest to smallest. Scaffold 7 is unplaced. **C.** Conserved macrosynteny with variable microsynteny comparisons of *C. sp. 61* to *C. elegans* . **D.** *Caenorhabditis* phylogeny of Elegans Group, with *C. japonica* as the outgroup species. **E.** Nigon classification of JU4110 chromosomes. The right y-axis shows the count of single-copy orthologs between JU4110 and *C. elegans* genes previously assigned to Nigon elements (A-E, N, X), grouped in 100 kb bins. **Table 1.** Properties of *Caenorhabditis* *sp. 61*

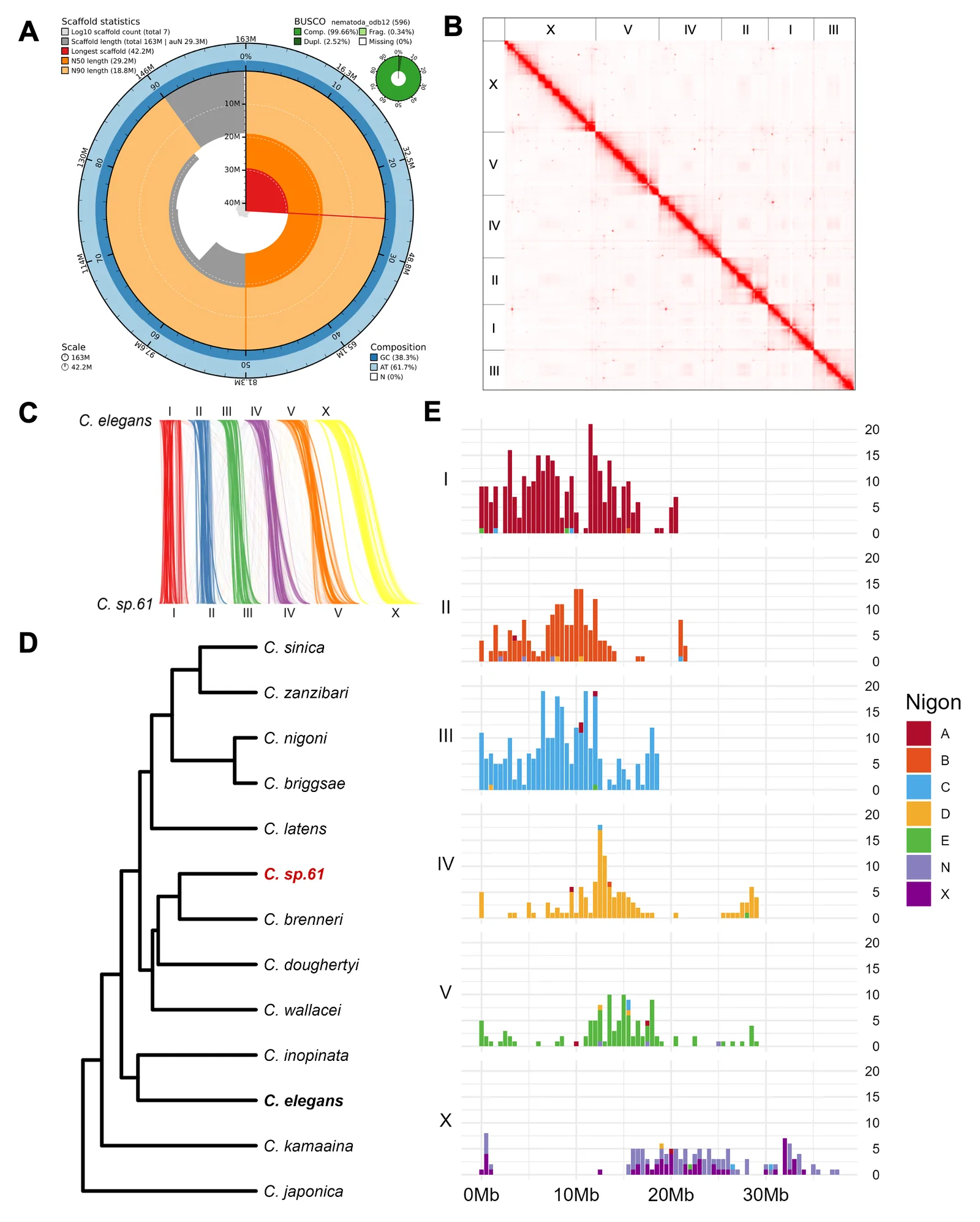



**Table:** 

Property	Metric
Location found	Sapa, Vietnam
Location coordinates	22.33947, 103.86148
Mating system	Dioecious
Genome size (Mb)	163
Approximate coverage (x) – PacBio HiFi	119
Approximate coverage (x) – Illumina Hi-C	389
Number of reads (Gb) – PacBio HiFi	19.4
Number of reads (Gb) – Illumina Hi-C	63.4
GC content (%)	38.3
Number of protein coding genes	19,780
Number of gaps	18
Repeats (%) – RepeatMasker	21.4
Repeats (%) – EarlGrey	26.5
SRA accession no. – PacBio HiFi	SRR38755789
SRA accession no. – Illumina Hi-C	SRR38755790
Isolated by	Marie-Anne Félix lab

## Description

Despite the continually expanding genome resources for the Rhabditidae family of nematodes, some recently discovered species do not have chromosome-level assemblies, including *Caenorhabditis* *sp.* *61* . Strain JU4110, also known as A325, is a laboratory-derived, highly inbred line of *Caenorhabditis* *sp.* *61,* produced from JU4045 through 25 rounds of single L4 female × single male crosses. JU4045 was originally isolated by the Marie-Anne Félix lab from a rotting flower near Sapa, Vietnam on November 30, 2019, and is characterized as a dioecious species (Table 1). Phylogenetically, *C. sp. 61* falls within the Elegans Group as a sister species to *C. brenneri* ( Figure 1, panel D) *.*

Previous studies of *Caenorhabditis* genome evolution have revealed significant conservation of chromosomal synteny despite large sequence divergence and frequent intrachromosomal rearrangements (Bouvarel et al., 2024; Thomas, 2008). Moreover, the addition of novel genome assemblies of *Caenorhabditis* improves phylogenomic coverage and enables more comprehensive comparative analyses of *Caenorhabditis* genome evolution. Here, we present a chromosome-level genome assembly for *C. sp. 61* , combining Pacific Biosystems (PacBio) HiFi long-read sequencing with Hi-C scaffolding (Figure 1).

The assembly was created with Hifiasm (Cheng et al., 2021), generating a 201 Mb haploid assembly composed of 200 contigs with a BUSCO completeness of 99.7% and N50 of 11.9 Mb. Subsequence removal of contaminant contigs with BLAST (Camacho et al., 2009), purging haplotigs with purge_dups (Li, 2018), and scaffolding with YaHS (Zhou et al., 2023) resulted in a final assembly made up of seven scaffolds that totaled 163 Mb in size and had an N50 of 29.2 Mb. The BUSCO results remained unchanged ( Figure 1, panel A). The seven scaffolds correspond to the expected six chromosomes which are highly conserved across *Caenorhabditis* , and one unplaced scaffold 173,000 bps in length ( Figure 1, panel B). Nucleotide BLAST reveals that the unplaced scaffold has similarity to *C. elegans* , *C. nigoni,* and *C. briggsae* segments. Annotation revealed that the *C. sp. 61* assembly contained 19,780 genes, making up 26.9% of the genome. BUSCO completeness of the gene annotations was 99.3%. Gene content between *C. sp. 61* and *C.* *elegans* is highly maintained with 6,789 single copy orthologs between the species (OrthoFinder v2.5.5; Emms & Kelly, 2019).

Scaffolding of the assembly showed that chromosomes I-V and X contain telomeric-repeat motifs (TRMs). Telomeres are composed of tandem arrays of TRMs, greater than 1 kb in length, that are recognized by telomere-binding proteins that ensure replication and protection of chromosomal ends. TRMs are conserved in Nematoda, with the canonical TRM sequence of TTAGGC (Lim et al., 2023). We found that each chromosome in the JU4110 assembly (except chromosome I) contains a variation of the TRM T A AG C C at the 5’ terminus. At the 3’ terminus, only chromosomes I-IV contain the canonical sequence TTAGGC exactly. The unplaced scaffold appears to have a repeat sequence at the 5’ terminus of the scaffold, but it does not match the canonical TRM.

*Caenorhabditis* genome evolution often shows conserved macrosynteny with variable microsynteny, meaning that broad chromosome architecture is generally consistent while gene content within a chromosome appears scrambled. This pattern holds in comparisons of *C. sp. 61* to *C. elegans* . All six chromosomes are conserved, along with the underlying Nigon element assignments. Nigon elements are putative ancestral chromosome linkage groups largely conserved through nematode evolution (Blaxter et al., 2024). Previous work by Rödelsperger (2024) shows that most large chromosome changes in nematodes may be explained by fusions and rearrangements of these Nigon elements, which can help trace chromosome evolution and species-specific changes. The characteristic distribution of gene and repeat density across each chromosome are also conserved – repeat density is elevated on chromosome arms relative to centers, while coding density shows the inverse pattern. However, numerous intrachromosomal rearrangements, inversions, and duplications disrupt local gene order ( Figure 1, panels C and E).

Mating system is also expected to impact repeat content in genomes, particularly on the sex chromosomes. Theoretically, effective population size of the X chromosome is three-fourths that of the autosomes in dioecious species, but equal to that of autosomes in androdiecious species. Thus, we would expect to see selection to be weaker on the X in dioecious species and therefore an expansion of repeats. The *C. sp. 61* assembly was annotated by EarlGrey (Baril et al., 2024) to contain 26.5% repeats, slightly higher than *C. elegans* at 20.5%. Across chromosomes, *C. sp. 61* has 3-4% more repeats than *C. elegans* , except for chromosome 3, which has an equal proportion of repeat content, and the X chromosome, which has twice the density of repeats than *C. elegans* (31.3% vs 14.1%). But comparisons of *C. brenneri* , the sister species of *C. sp. 61* , and another dioecious *Caenorhabditis* , reveal similar repeat content to *C.* *elegans* with 20.9% of the genome and 15.2% of the X chromosome made up of repeats. The type of repeats differ between the genomes as well. *Caenorhabditis* species repeat content is primarily type 2 transposable elements (TEs). For example, of the 20.5% repeats, roughly half (9.47%) is DNA TEs. Of the 26.5% repeats of *C. sp. 61* , only 4.48% is DNA TEs. Rather, an elevated level of long terminal repeats (LTRs) (2.30%) and rolling circle (3.20%) TEs appear to determine the repeat landscape of *C. sp. 61* . Analysis of Kimura distance supports this. Kimura distance, often used as a proxy for TE age based on sequence similarity, shows steady degradation of *C.* *elegans* N2 repeats, specifically type 2 TEs, but *C. sp. 61* seems to have a “recent burst”, or repeats with greater sequence identity, especially in LTRs. Overall, the repeat content of *C. sp. 61* is expanded, particularly on the X and with LTRs, showing species-specific patterns which will contribute to our broader understanding of the evolutionary forces shaping nematode genomes.

## Methods

**Collection:** Prior to transfer to our research group, JU4110 remained cryopreserved. Standard *Caenorhabditis elegans* maintenance protocols were used to culture JU4110 populations on agar plates made from nematode growth media at 20°C, seeded with OP50 strain of *Escherichia coli* . To expand populations for DNA extractions, worms were transferred using a "chunk" of agar to three 100 mm plates seeded with *E. coli* and were left to incubate at 20°C for 2-3 days. Mixed-age worms were washed off with M9 buffer into a 15 mL conical tube and then washed twice with M9 buffer to reduce surface contaminants. Washed worms were finally resuspended in 10 mL M9 and remained on a rocker overnight (~17 hours) to expel potential contaminants in the gut. Before extraction, two additional M9 washes were performed, worm bodies were isolated by centrifugation, the supernatant discarded, and the resulting pellet aliquoted into 50 µL volumes in 1.5 mL tubes.

**Long-read sequencing:** Promega Wizard® HMW DNA Extraction Kit (cat. no. A2920) was used for DNA long-read sequencing using the manufacturer's protocol with minor modifications. Worm cuticles were disrupted through routine freeze/thaw cycles, alternating between −80°C for five minutes and 37°C until thawed, with brief vortexing between cycles, repeated five times. All centrifugation steps were performed at 4°C, and alcohols were kept on ice until required. An additional incubation of 25 minutes at 65°C was incorporated at the lysis step. Samples were subsequently given to the University of Miami John P. Hussman Institute for Human Genomics Sequencing Core Facility (RRID:SCR_017828) for PacBio sequencing.

**Hi-C sequencing:** Extra tubes of 50 µL worm pellet were frozen at −80°C using a Mr. Frosty™ freezing container (Thermo Scientific cat. no. 5100-001) filled with 100% isopropanol to prevent ice crystal formation. Two tubes were then mailed on dry ice to Arima Genomics for High Coverage Chromatin Conformation Capture sequencing (Hi-C).

**Genome Assembly:** Genome assembly was performed using Hifiasm v0.16.0 (Cheng et al., 2021) with default parameters and PacBio HiFi and Arima Hi-C libraries. Contaminant contigs were later identified and discarded from both the diploid and phased haploid assemblies using BLAST v2.14.1 (Camacho et al., 2009). To identify duplications and remove alternative haplotypes, PacBio HiFi reads were mapped back to the assembly with minimap2 v2.30 using parameters -xasm5 -DP (Guan et al., 2020), after which read depth cutoffs were set manually using the -l 90 -m 114 -u 130 settings in purge_dups v1.2.6 (Li, 2018). At each step, assembly quality was assessed using QUAST v5.3.0 (Gurevich et al., 2013) with default parameters and BUSCO v6.0.0 (Manni et al., 2021) run against the Nematoda odb12 lineage dataset with options -m genome and --offline.

**Phylogenetic Analysis:** A multigene tree was calculated from the BUSCO output of 95 species within Rhabditidae. Single copy orthologs were concatenated for multiple sequence alignment, which was completed with MAFFT v.7.221 using the localpair option with 1000 times iterative refinement (Katoh and Standley, 2013). Alignments were trimmed using ClipKIT v.2.3.0 with option smart-gap (Steenwyk et al., 2020) and partitioned with AMAS concat (Borowiec, 2016). IQ-TREE v.1.6.12 was used to compute the phylogenetic tree with option -m MFP (Nguyen et al., 2015). Branch length supports were calculated with SH-like approximate likelihood ratio test and 1000 bootstrap replicates (Guindon et al., 2010). Visualization was done in RStudio with package phytools (Revell, 2012). Tips containing species too distant from *C. sp. 61* were dropped for clarity.

**Hi-C Mapping:** Hi-C raw data was processed and aligned with Juicer v2.0 (Durand et al., 2016) using default parameters and assembled using the --assembly option. We used YaHS v1.2.2 (Zhou et al., 2023) for scaffolding and Juicebox v2.3.6 (Robinson et al., 2018) for visualization and assessment.

**Gene and Repeat Annotation:** Genomes were first softmasked with RepeatModeler2 (Guan et al., 2020) and RepeatMasker (Smit et al., 2013), while RNA reads were aligned to the genome using STAR v2.6.1a with the --outSAMstrandField intronMotif option (Dobin et al., 2013). Genome annotation was subsequently performed using BRAKER3 v3.0.8 (Gabriel et al., 2024) on the softmasked assemblies using the Nematoda odb10 protein dataset with RNA sequence data from NCBI project PRJNA1256413 (O’Leary et al., 2024). BRAKER3 depends on GeneMark (unsupervised) and AUGUSTUS (supervised), two generalized hidden Markov models for gene prediction (Bruna et al., 2024; Wong & Simakov, 2019; Stanke et al., 2006), with resulting protein sets consolidated by TSEBRA (Gabriel et al., 2021) to maximize BUSCO completeness. We subsequently filtered protein predictions for the longest isoform using AGAT v1.4.1 (Dainat et al., 2026) via the scripts agat_sp_keep_longest_isoform.pl and agat_sp_extract_sequences.pl, and assembly statistics were generated with agat_sp_statistics.pl. OrthoFinder v2.5.5 (Emms & Kelly, 2019) was used to find single copy orthologs between *C. sp. 61* and *C. elegans.* The six major chromosomes were identified by location of single copy orthologs on *C. elegans* chromosomes *.* Nigon elements classifications were assigned to single copy orthologs using a list of known gene:Nigon associations from Gabriel et al. (2021).

**Snail Plot** : We used BlobTk v0.8.0 (Challis & Blaxter, 2026) to produce the snail plot with the soft-masked assembly. BUSCO v6.0.0 (Manni et al., 2021) and nematoda_odb12 dataset helped recalculate assembly BUSCO scores.

**Data availability**

Bioinformatic scripts, workflows and software commands are available at https://github.com/jannafierst/HiC_Assemblies

Genome and annotation files are available on Zenodo at https://zenodo.org/records/21996893

**Nucleotide sequence accession numbers**

The complete genome sequence is available at NCBI under Bioproject PRJNA1256413. DNA libraries used in this project have been deposited at the Sequence Read Archive (SRA) under SRR38755790 (Hi-C) and SRR38755789 (PacBio).
